# Discovery of *Leptospira* spp. seroreactive peptides using ORFeome phage display

**DOI:** 10.1371/journal.pntd.0007131

**Published:** 2019-01-24

**Authors:** Siti Roszilawati Ramli, Gustavo M. S. G. Moreira, Jonas Zantow, Marga G. A. Goris, Van Kinh Nguyen, Natalia Novoselova, Frank Pessler, Michael Hust

**Affiliations:** 1 Research Group Biomarkers for Infectious Diseases, Helmholtz Centre for Infection Research, Braunschweig, Germany; 2 Institute for Biochemistry, Biotechnology and Bioinformatics, Braunschweig University of Technology, Braunschweig, Germany; 3 Institute for Medical Research, Kuala Lumpur, Malaysia; 4 OIE and National Collaborating Centre for Reference and Research on Leptospirosis Academic Medical Center, Department of Medical Microbiology, University of Amsterdam, Amsterdam, the Netherlands; 5 Research Group Biomarkers for Infectious Diseases, TWINCORE Center for Experimental and Clinical Infection Research, Hannover, Germany; 6 United Institute of Informatics Problems, National Academy of Sciences of Belarus, Minsk, Belarus; Medical College of Wisconsin, UNITED STATES

## Abstract

**Background:**

Leptospirosis is the most common zoonotic disease worldwide. The diagnostic performance of a serological test for human leptospirosis is mainly influenced by the antigen used in the test assay. An ideal serological test should cover all serovars of pathogenic leptospires with high sensitivity and specificity and use reagents that are relatively inexpensive to produce and can be used in tropical climates. Peptide-based tests fulfil at least the latter two requirements, and ORFeome phage display has been successfully used to identify immunogenic peptides from other pathogens.

**Methodology/Principal findings:**

Two ORFeome phage display libraries of the entire *Leptospira spp*. genomes from five local strains isolated in Malaysia and seven WHO reference strains were constructed. Subsequently, 18 unique *Leptospira* peptides were identified in a screen using a pool of sera from patients with acute leptospirosis. Five of these were validated by titration ELISA using different pools of patient or control sera. The diagnostic performance of these five peptides was then assessed against 16 individual sera from patients with acute leptospirosis and 16 healthy donors and was compared to that of two recombinant reference proteins from *L*. *interrogans*. This analysis revealed two peptides (SIR16-D1 and SIR16-H1) from the local isolates with good accuracy for the detection of acute leptospirosis (area under the ROC curve: 0.86 and 0.78, respectively; sensitivity: 0.88 and 0.94; specificity: 0.81 and 0.69), which was close to that of the reference proteins LipL32 and Loa22 (area under the ROC curve: 0.91 and 0.80; sensitivity: 0.94 and 0.81; specificity: 0.75 and 0.75).

**Conclusions/Significance:**

This analysis lends further support for using ORFeome phage display to identify pathogen-associated immunogenic peptides, and it suggests that this technique holds promise for the development of peptide-based diagnostics for leptospirosis and, possibly, of vaccines against this pathogen.

## Author summary

Leptospirosis is an infectious disease that is transmitted from animals to humans. It is associated with a broad range of clinical presentations, and diagnostic tests with high diagnostic accuracy are required in order to enable accurate diagnosis. Leptospirosis is diagnosed by detecting DNA of the pathogen or antibodies against it in patients’ blood; the latter are preferred in resource limited regions, and diagnostics based on peptides (small fragments of proteins) are advantageous because they are inexpensive to produce and more stable in hot climates than full-length proteins. We used a technique called open reading frame phage display to identify peptides from *Leptospira spp*. that could be used to detect antibodies against them in human blood. In this method, the pathogen’s genome is fragmented, the corresponding peptides displayed on the surfaces of phages (viruses that infect bacteria), and the peptides that bind most strongly to the patients’ antibodies are then selected by screening. Using this method, we identified 2 leptospiral peptides that accurately identified antibodies against *Leptospira spp*. in sera from patients with leptospirosis. These results are encouraging because they demonstrate that ORFeome phage display may be a powerful tool to develop better diagnostics for leptospirosis for use in less developed areas.

## Introduction

Leptospirosis is the most common and geographically widespread zoonotic disease worldwide. It is caused by pathogenic strains of the spirochete *Leptospira spp*. Humans are accidental hosts who get infected through direct contact with body fluids of carrier animals or with contaminated water and soil [1]

Human leptospirosis is a major problem in countries of tropical and, less often, subtropical climates. Numerous outbreaks have been reported in association with rainy seasons, floods, and recreational and sports activities [2]. Globally, leptospirosis is estimated to occur at a frequency of approximately 1.03 million cases per year with a mortality of 5.7% [3]. In Malaysia, incidence is estimated to be 1–10 cases per 100,000 individuals per year with case fatality rates around 10% [4]. Leptospirosis was designated a notifiable disease in 2011, and reported incidence increased subsequently, as illustrated by a reported incidence for 2015 of 17.5 cases per 100,000 individuals, 86 outbreaks, and case fatality rates of 0.6% [5].

The microscopic agglutination test (MAT) is the serological ‘gold standard’ for diagnosis of leptospirosis. Unfortunately, this test is not only time-consuming and laborious, but it also can only be performed by a reference laboratory with a collection of serovars endemic in the region. To overcome the drawbacks of the MAT, numerous serological assays have been developed that do not depend on the availability of viable leptospires, particularly IgM ELISA tests based on either whole cell extracts or recombinant proteins [6–8]. Other serological approaches that have been described were agglutination, dipstick, and lateral flow assays [8]. Among these serological tests, in-house Leptospira ELISA using whole cell extracts have achieved the highest specificity and sensitivity [9]. Nevertheless, this method requires the standardized preparation of whole cell extracts from serovars most relevant to the geographical region [10].

Phage display is mainly used for antibody generation [11], but also for the identification of pathogen-associated immunogenic proteins; for the latter, it is based on using fragmented pathogen genomes or cDNA fragments to allow high-throughput screening for novel potential antigens [12–16]. Immunogenic proteins are often identified using 2D-PAGE of proteins from cultivated bacteria followed by mass spectrometry. However, phage display offers clear advantages, notably better performance for the identification of immunogenic proteins below 10 kD, low abundance proteins, and proteins only expressed in host-pathogen interaction [17, 18]. ORFeome phage display is an improved variant of the phage display technique in which the library quality of fragmented genomes or cDNA fragments is improved by a unique open reading frame enrichment step [19–22].

Diagnostics for use in hot, resource-limiting environments should be inexpensive to produce and stable at ambient temperature. Peptide antigens fulfil both requirements; besides, they offer the added advantage that they can be synthesized in biotinylated form, thus facilitating their use in streptavidin-based diagnostics.

In the present study, we have used ORFeome phage display to identify *L*. *interrogans* peptides that are immunogenic in humans and can be used to develop relatively inexpensive diagnostics tools for this challenging pathogen.

## Methods

### Ethics statement

The study protocol NMRR-14-1687-23346(IIR) involved the use of patients’ sera obtained for clinically indicated diagnostics to be used for research purposes and was approved by the Medical Research & Ethics Committee, Ministry of Health Malaysia, Malaysia. All human biosamples were anonymized.

### Patients and serum samples

Sera from 34 patients who presented in the year 2015 with symptoms suggestive of acute leptospirosis were obtained from the Public Health Laboratory of Kota Bharu and Kota Kinabalu, Malaysia. The sera were obtained from hospitalized patients after an average of three to seven days of illness and had MAT titers of 1:800, which was consistent with the acute phase of leptospirosis. Infection with other pathogens, including Dengue virus, was excluded by the respective diagnostics as suggested by clinical history and other laboratory findings. Dengue fever is the most common infectious disease in this region and it may cause false positive results in *Leptospira* spp. ELISA tests due to cross-reactivity. All samples were therefore tested for the Dengue virus NS1 protein and anti-Dengue IgM and IgG reactivity and were thus ensured to be negative.

The patients presented with fever and one or more of these signs and symptoms: headache, myalgia, arthralgia, conjunctival injection, anuria or oliguria and/or proteinuria, jaundice, pulmonary and/or intestinal hemorrhage, cardiac arrhythmia or failure, skin rash, and gastrointestinal symptoms such as nausea, vomiting, abdominal pain, and diarrhea.

Sera from 18 patients were pooled into two groups based on their reactivity to Malaysian strains (n = 8) or WHO reference strains (n = 10). Two sets of *Leptospira*-negative control sera were used. The control pool used in S2 Fig was obtained from 7 healthy adult volunteers from Germany with no travel history to *Leptospira*-endemic countries. The other 16 patient sera were used for the titration ELISAs in the ORFeome procedure. The individual control sera for ELISA validation shown in Fig 2A were obtained from 16 healthy adults of Caucasian origin who participated in an unrelated epidemiological study [23]. Absence of leptospiral seroreactivity in the control sera was confirmed by in-house ELISA with leptospiral culture antigens.

### Construction of *Leptospira* spp. genomic DNA library

Two libraries of *Leptospira spp*. were constructed using genomic DNA. The first library consisted of five strains of *L*. *interrogans* isolated from leptospirosis patients in Malaysia between 2014 and 2015. The second library consisted of seven WHO reference strains which were obtained from the Leptospirosis Reference Centre (also known as OIE Reference Laboratory for Leptospirosis, Amsterdam Medical Centre, Amsterdam). The strains are listed in Table 1. Strains from both groups were cultured in Ellinghausen-McCullough-Johnson-Harris (EMJH) medium at 30°C for 7–10 days at 250 rpm. Genomic DNA was isolated from pellets of 5 mL culture centrifuged at 8000 x g for 30 minutes (min), using the QiaAmp DNA Mini Kit according to the manufacturer’s instructions (Qiagen, Hilden, Germany). The extracted DNA for each library was mixed and amplified with the illustra™ Ready-To-Go GenomiPhi V3 DNA amplification kit (GE Healthcare) according to the manufacturer’s instructions. Twenty μg of DNA from each mixed and amplified genomic library were fragmented by sonication upon extraction. Subsequently, the DNA was concentrated using Amicon Ultra 0.5 mL centrifugal filters with a cut-off of 30 kDa.

**Table 1:** List of strains used to construct the two *Leptospira spp*. genomic libraries.

Genomic Library	Species	Serogroup	Serovar	Strain
I	*L*. *interrogans*	Canicola	Bindjei	782
	*L*. *interrogans*	Ictrohaemorrhagiae	Copenhageni	898
	*L*. *interrogans*	Bataviae	Paidjan	1489
	*L*. *interrogans*	Bataviae	Losbanos	1548
	*L*. *interrogans*	Icterohaemorrhagiae	Smithi	1530
II	*L*. *interrogans*	Australis	Australis	Ballico
	*L*. *interrogans*	Bataviae	Bataviae	Swart
	*L*. *weillei*	Celledoni	Celledoni	Celledoni
	*L*. *kirschneri*	Grippotyphosa	Grippotyphosa	Duyster
	*L*. *interrogans*	Sejroe	Hardjoprajitno	Hardjoprajitno
	*L*. *borgpetersenii*	Javanica	Javanica	Veldrat Batavia 46
	*L*. *biflexia*	Semaranga	Patoc	Patoc 1

DNA fragments with sizes from 100 to 800 bp were extracted from an agarose gel and the DNA ends were repaired with the Fast DNA End Repair Kit (Thermo Scientific) according to the manufacturer’s instructions. 1.4 μg of fragmented DNA were then ligated into 1.4 μg of the *Pme*I-digested pHORF3 vector [20] and subsequently transformed into *E*. *coli* TOP10F’ (Invitrogen) by electroporation. Colony PCR was performed in some of the resulting clones to determine the insert rate of ligation.

### Packaging of a phage display library with hyperphage

The library was packaged using Hyperphage [24, 25] as described before [19, 20]. By packaging the genomic DNA library with Hyperphage, ORFs are enriched and the resulting oligopeptides are presented on the phage particles for panning. The *E*. *coli* XL1-Blue MRF’ containing the library was inoculated into 400 mL 2x YT-GA medium (2x yeast-tryptone broth supplemented with 0.1 M glucose and 100 μg/mL ampicillin) to an OD_600_ <0.1 and grown at 37°C, 250 RPM until OD_600_ ≈0.5. At this point, the culture was infected with Hyperphage (MOI 1:20) for 30 min at 37°C without shaking, and then 30 min under 250 RPM.

The culture was then centrifuged, suspended in 400 mL 2x YT-AK medium (2x YT containing 100 μg/mL ampicillin and 50 μg/mL kanamycin), and phage particles were produced at 30°C and 250 rpm overnight. Cells were then centrifuged for 20 min at 10,000 x g, and phage particles in the supernatant were precipitated with 1/5 volume of polyethylene glycol (PEG)/NaCl solution (20% w/v PEG 6000), 2.5 M NaCl) for 3 hours (h) on ice with gentle shaking. Phage particles were then pelleted for 1 h at 10,000 x g and suspended in 10 mL phage dilution buffer (10 mM TrisHCl pH 7.5, 20 mM NaCl, 2 mM EDTA). Remaining bacteria were pelleted by an additional centrifugation step of 10 min at 20,000 x g, and the solution was then filtered through a 0.45 μm filter to remove residual bacteria. The filtrate was again precipitated with 1/5 PEG/NaCl for 1 h and then centrifuged for 30 min at 20,000 x g. Pellets were suspended in 1 mL phage dilution buffer and residual bacteria removed by centrifugation for 1 min at 16,000 x g. The final supernatant containing the oligopeptide presenting phages was stored at 4°C. Phage titers were determined as described previously [26].

### Library validation by colony PCR and sequencing

To check library quality, a number of random *E*. *coli* colonies were analyzed by colony PCR and sequenced after packaging with Hyperphage. Therefore, the primers MHLacZPro_f (5'-GGCTCGTATGTTGTGTGG-3') and MHgIII_r (5'-GGAAAGACGACAAAACTTTAG-3') were used with the following PCR protocol: 98°C 30 s, 98°C 10 s, 56°C 20 s (35 cycles), 72°C 60 s and a final extension of 72°C for 2 min. The DNA was separated and analyzed by gel electrophoresis (Qiaxcel Advanced). Additionally, plasmid DNA was sequenced with the primers used for colony PCR to verify the correct inserts and the ORF enrichment after packaging.

### Selection of immunogenic oligopeptide phage by panning

Two wells of a Maxisorp™ 96 well microplate were coated with 150 μL of goat IgG directed against human IgG, IgA and IgM Fc (Dianova 109-005-064, 2 mg/mL, Lot No. 113036) diluted in 1:500 phosphate buffered saline (PBS) and another six wells were coated with 5 x 10^10^ CFU Hyperphage in PBS and incubated at 4°C overnight. Subsequently, the coating solutions were removed and the wells were blocked for 30 min with PBS with 0.1% Tween, supplemented with 2% (w/v) milk powder (2% MPBST).

A pool of twelve patients’ sera was diluted 1:100 in 2% MPBST and pre incubated twice in the Hyperphage coated wells for 1 h to eliminate IgG binding to helper phage. After pre-incubation, the serum pool was incubated for 1.5 h in the wells coated with goat anti-human IgG, A, M antibody. The *Leptospira spp*. phage library (corresponding to 1.1 x 10^10^ CFU) was mixed 1:3 with 2% MPBS-T and incubated in the wells with the pooled leptospirosis patients’ sera for 1.5 h. Unbound phage and phage with low affinity were removed by stringent washing steps. Three panning rounds were performed, and the wells were washed twice after each step, with one additional wash for the second panning round. After washing, bound phage particles were eluted with 200 μL of 10 μg/mL trypsin in PBS for 30 min at 37°C. Eluted phages of both wells were combined and 10 μL were used for titration. The remaining 390 μL were used to infect 20 mL of an *E*. *coli* TOP10F’ culture grown to an OD_600_ of 0.5. The cells were incubated for 30 min at 37°C and harvested by centrifugation for 10 min at 3250 x g. The pellet was suspended in 250 μL 2xYT-GA. The bacterial suspension was plated onto 15 cm 2xYT-GA agar plates and incubated overnight at 37°C. Colonies were swept off with 5 mL 2xYT-GA medium, then 50 mL of 2xYT-GA medium was inoculated with the bacterial suspension to an OD_600_ of <0.1 and grown to an OD_600_ of 0.5 at 37°C and 250 rpm. For infection, 5 mL of the bacterial culture (approx. 2.5 x 10^9^ cells) was mixed with Hyperphage infected at an MOI of 1:20 resulting in 5 x 10^10^ CFU Hyperphage. The suspension was incubated at 37°C for 30 min without shaking and another 30 min at 37°C and 250 rpm. To remove glucose, which inhibits phage expression, the infected cells were harvested by centrifugation for 10 min at 3220 x g. The remaining pellet was resuspended in 30 mL 2x YT-AK and incubated at 30°C and 250 rpm overnight for phage production. The bacterial cells were then pelleted by centrifugation for 20 min at 3220 x g and the remaining supernatant was used to precipitate phage particles with PEG/NaCl (20% (w/v) PEG 6000, 2.5 M NaCl). Thirty mL of supernatant were separated and incubated for 1 h with 6 mL PEG/NaCl on ice with slight shaking on a rocker, followed by centrifugation at 6000 x g for 1 h at 4°C. The phage pellet was resuspended in 500 μL phage dilution buffer (10 mM TrisHCl pH 7.5, 20 mM NaCl, 2 mM EDTA), centrifuged in a microcentrifuge at 16,100 x g for 1 min and the supernatant was used for further panning rounds. For the 2^nd^ and 3^rd^ panning rounds, 150 μL of the amplified phage was used. Eluted phage particles from the 3^rd^ panning round were used for titration without further amplification. Single colonies were then used for single oligopeptide phage production.

### Production of single oligopeptide phage clones for screening

Single oligopeptide phage clones were produced by inoculating 175 μL 2x YT-GA medium with single colonies from the titration plate in a polypropylene 96-well U-bottom plate (Greiner bio-one). The cultures were incubated at 37°C and 500 rpm shaking overnight.

From this plate, 10 μL were used to inoculate another 165 μL 2xYT-GA medium per well, which was incubated at 37°C and 800 rpm for 2 h. Subsequently, the bacteria were infected with 5 x 10^9^ cfu Hyperphage and incubated for 30 min at 37°C without shaking and 30 min at 37°C and 800 rpm. The bacteria were pelleted by centrifugation at 3220 x g for 10 min and the pellets were resuspended in 175 μL/well 2x YT-AK and incubated overnight at 30°C and 800 rpm. The produced phage in the supernatant were transferred to another plate and precipitated with 1/5 volume of PEG/NaCl solution for 1 h at 4°C. Next, precipitated phage particles were pelleted by centrifugation at 3220 x g for 1 h and the pellets dissolved in 150 μL PBS. Remaining bacterial cells were separated by another centrifugation step and the phage-containing supernatants stored in a new plate at 4°C and used for screening ELISA.

### Screening ELISA of oligopeptide phage clones

Two types of ELISA were performed for selection of oligopeptide phage clones. First, a screening ELISA was performed on each genomic library. Oligopeptide phage particles were captured by a monoclonal mouse anti-M13 (B62-FE2, Progen) antibody for screening. For this, 100 μL of a 250 ng/mL solution of antibody in PBS were coated overnight at 4°C and subsequently blocked with 2% MPBST. The wells were washed after each incubation step three times with 300 μL PBST. One hundred μL of the monoclonal phage clones were added to each well and incubated for 2 h at 4°C. 100 μL of pooled patients’ sera reactive to Malaysian strains were added to library 1 while pooled sera reactive to WHO strains were added to library 2. All sera were diluted in 2% MPBST supplemented with 10% *E*. *coli* TOP10F’ lysate and 1 x 10^10^ CFU/mL Hyperphage. The dilutions were incubated at RT for 2 h prior to use in the ELISA. Then, the dilutions were added onto the captured phage particles for 1.5 h and detected via a goat anti-human IgG, A, M antibody conjugated to horseradish peroxidase (HRP) (1:20,000) for 1.5 h. Visualization was achieved by adding 100 μL TMB (3,3’,5,5’-tetramethylbenzidine) solution and the reaction was stopped with 100 μL 1 N sulfuric acid. A SUNRISE microtiter plate reader (Tecan, Crailsheim, Germany) was used to measure absorbance at 450 nm and subtract scattered light at 620 nm.

A second titration ELISA was performed on the selected unique oligopeptide clones from the screening ELISA. The method was the same except this time clones were tested for reactivity against each of three aforementioned serum pools. The sera were serially diluted 2-fold from 1:100 to 1:102,400 in 2% MPBST supplemented with 10% *E*. *coli* TOP10F’ lysate and 1 x 10^10^ CFU/mL Hyperphage.

### Production of control proteins and synthetic peptide

The *lipL32* and *loa22* genes were amplified from *L*. *interrogans* serovar Copenhageni genomic DNA. Phusion DNA Polymerase (Thermo Scientific F-530L) was used to amplify full-length genes according to the following protocol: 98°C 30 seconds (s), 98°C 10 s, annealing temperature primer dependent 20 s, 72°C 20 s, 30 cycles, 72°C 10 min. The amplified genes were digested with Nde1 and Not1 and the resulting fragments resolved by 1% agarose electrophoresis, purified from the gel with NucleoSpin Gel and PCR Clean-Up kit (Macherey-Nagel 740609.250), ligated into the Nde1/Not1 digested vector pET21a(+) and the ligation product subsequently transformed into *E*. *coli* BLR (-DE3). Finally, positive clones were identified by colony PCR and confirmed by sequencing.

For protein expression, 200 mL 2xYT-GA medium were inoculated with 10 mL overnight culture and incubated at 37°C and 120 rpm in a baffled flask to an OD600 of 0.6. Expression was induced with a final concentration of 1 mM IPTG for 6 h at 30°C, followed by centrifugation at 3,000 x g for 20 min for cell harvesting. Cells were then suspended in His-tag binding buffer pH 8 with urea (50 mM Na2HPO4, 100 mM NaCl, 10 mM imidazol, 8 M urea) and incubated for 1 h under over-head rotation, followed by sonication (6 cycles of 10 s 50% power, 10s incubation on ice, Sonotrode MS72, Bandelin). Subsequently 0.5 mL Ni-NTA agarose slurry (Qiagen 30210) was added to the disrupted cell solution and incubated for 1 h under over-head rotation. Then, the solution was loaded onto a polypropylene column. The agarose settled by gravity flow and was washed with 10 mM, 30 mM and 50 mM imidazole (50 mM Na2HPO4, 300 mM NaCl, 8 M urea, pH 8). Elution was achieved with 3 x 1.25 mL PBS pH 7.4 supplemented with 100 mM EDTA and 8 M urea.

The proteins were prepared for analysis by 12% SDS-PAGE by heating 0.5 μg of protein sample mixed with 5x Lane Marker Reducing Sample Buffer (Thermo Scientific 39000) at 95°C for 5 min. PageRuler Plus Prestained Ladder (Thermo Scientific 26619) and Spectra Multicolor Low Range Protein Ladder (Thermo Scientific 26628) were used as size marker. The samples were stacked for 10 min at 60 V, followed by separation for 60 min at 110 V. The gels were then stained with Coomassie Brilliant Blue R250 solution dye. The protein bands migrated at ~32 kDa and ~22 kDa.

*Leptospira spp*. whole-cell antigen for the whole-cell in house ELISA was prepared using the supernatant of live *Leptospira spp*. cultures. The antigen was prepared and coated to microtiter plates essentially as described by [27].

### ELISA for validation of immunogenicity of synthetic peptides

To confirm the interaction of each isolated peptide with the sera, 200 ng of synthetic peptide (Peps4LS GmbH, Heidelberg, Germany) was diluted in 100 μL of PBS and coated to a high binding 96-well microtiter plate (Greiner-bio one) and incubated at 4°C overnight. Blocking was performed with 2% MPBST for 30 min. Then, 16 sera from patients with acute leptospirosis (MAT titer, 1:800; reactive towards endemic serovars in Malaysia i.e. Australis, Autumnalis, Bataviae, Canicola, Celledoni, Copenhageni, Djasiman, Gryppotyphosa, Hardjobovis, Hardjoprajitno, Icterohaemorrhagiae, Javanica, Lai, Patoc, Terengganu, Sarawak) and 16 sera from healthy donors were serially diluted 2-fold from 1:100 to 1:102,400 in 2% MPBST and added into the wells for 1.5 h. After incubation, the wells were washed three times with 300 μL PBST. Bound IgM was detected with mouse IgG anti-human IgM (CH2)-HRPO, MinX none 100 μg; product no. AFC-5349-2, Dianova) diluted 1:20,000 in 2% MPBST, by incubation for 1.5 h at RT. The reaction was developed with TMB solution, stopped with sulfuric acid, and the plates were read at 450 nm as described before.

Similar ELISA steps were repeated with control proteins (rLipL32 and rLoa22) and *Leptospira* antigens. For ELISA of control proteins, the sera were diluted and titrated from 1:100 to 1:102,400 in 2% MPBST supplemented with 10% *E*. *coli* TOP10F’ lysate and 1 x 10^10^ CFU / mL Hyperphage and incubated for 2 h prior to use, as done in the ELISA of oligopeptide phage clones described above.

### Statistical analyses

Antigen/antibody ELISA signals in control and disease groups were non-normally distributed, and statistical significance of between-group differences was therefore assessed with the Wilcoxon rank sum (Mann-Whitney U) test [28]. To evaluate discriminatory biomarker potential, a logistic regression model fitted using Bayesian generalized linear models [29] was used to calculate the area under the receiver operating characteristic (ROC) curve (AUC). The AUC and corresponding confidence intervals (CI) values were estimated using the cross-validation procedure based on 1000 bootstrap samples as described before [30, 31]. In addition, multiple logistic regression was used to evaluate the classification performance of each combination of peptides / proteins.

## Results

### Construction of *Leptospira spp*. genomic phage display library

Genomic DNA of *Leptospira spp*. was fragmented by sonication and cloned into pHORF3, resulting in libraries with 3.0 x 10^7^ (Malaysian local strains library, i.e. library I) and 2.2 x 10^7^ (WHO strains library, i.e. library II) independent clones. The insert rates and sizes were analyzed by colony PCR and sequencing. Thirteen randomly picked clones of each library were used for colony PCR and an additional seven per library were picked for sequencing. The average insert size was 250 bp and 290 bp for library I and library II, respectively. All libraries had insert rates of more than 80%.

Both genomic libraries were then packaged with Hyperphage for the selection of open reading frames. The packaged libraries were checked for the number of inserts by colony PCR and for correct in-frame inserts by sequencing. The cloned libraries had an in-frame insert ratio of approximately 50 to 55% and phage titers of 2.9 x10^10^ and 4.0 x 10^10^ cfu/mL. The average DNA fragments were shorter compared to the initial size, i.e. 160 bp and 120 bp for library I and II, respectively.

### Panning and screening of oligopeptide phage

The pooled sera from patients with acute leptospirosis were used as polyclonal antibody source in the panning rounds and screening. Individual clones were picked after the second and third panning rounds during which interaction partners with low affinities were removed, thus selecting the best interaction partners. The results of this panning are summarized in S1 Fig.

**Fig 1:**
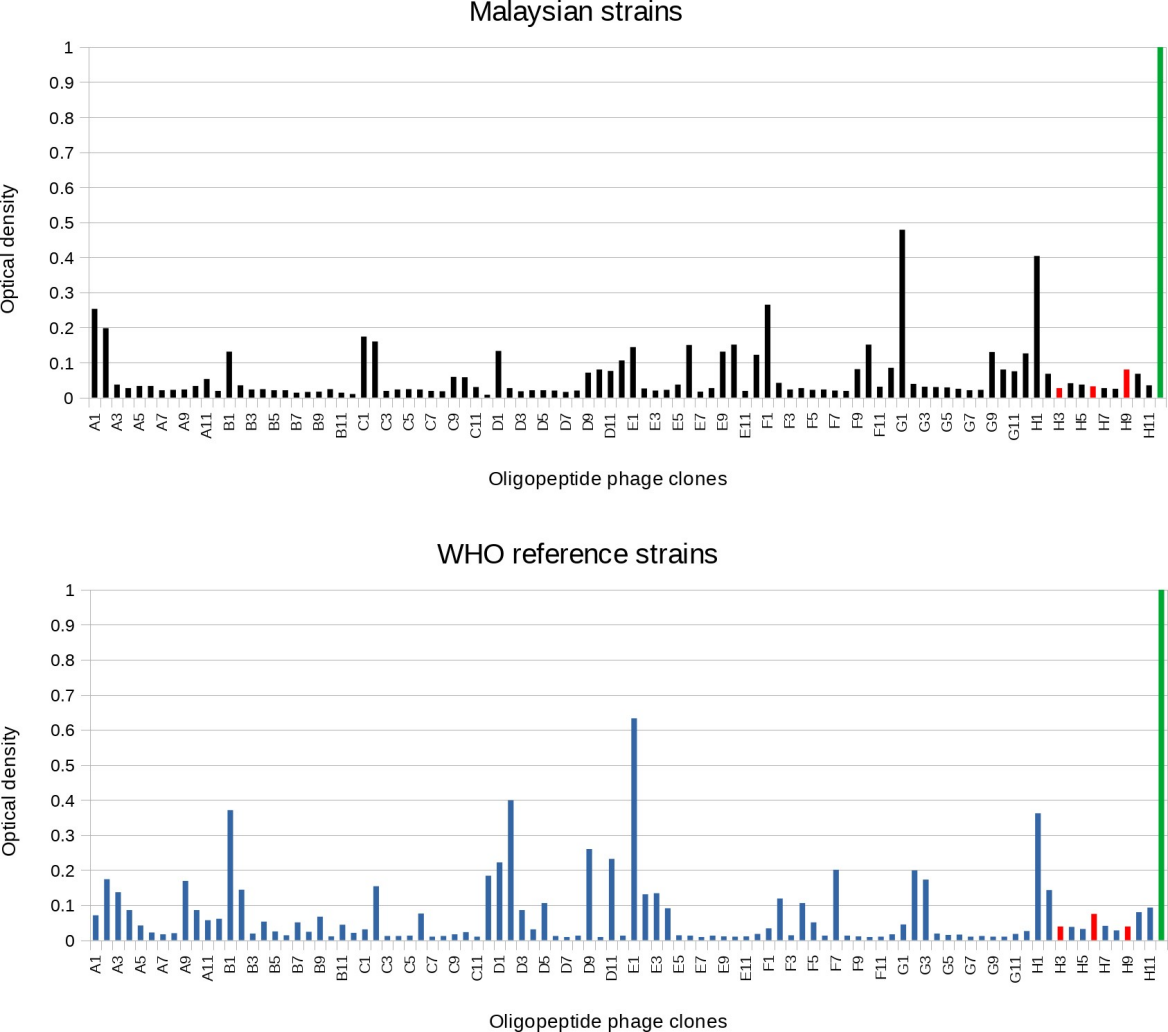
Screening ELISA of oligopeptide phage M13 clones. **(A) Malaysian strains (B) WHO reference strains.** Three negative controls (red) were included: medium alone (H3), captured phage displaying an irrelevant polypeptide (anti-lysozyme antibody; H6), captured Hyperphage (H9). Wells A1-H11 were incubated with pooled patient sera followed by detection with goat-anti human HRP conjugate. As positive control for phage production (green, H12), phage particles displaying anti-lysozyme antibody were captured and then detected directly with an anti-M13 HRP conjugate. Letter-number combinations on the x-axis represent the microtiter plate wells.

From both libraries combined, this resulted in the selection of 92 oligopeptide phage clones to be screened by ELISA. Of these, 35 had signals two-fold higher than the negative control and were analyzed further by sequencing (Fig 1). Eighteen of these 35 clones were identified as unique sequences and matched *Leptospira spp*. sequences according to BLAST analysis [32]. Their encoded amino acid sequences were translated using Expasy [33] and the corresponding *Leptospira spp*. proteins identified using BLAST (Table 2).

**Table 2:** BLAST results of the 18 unique oligopeptide phage clones. Summary of peptides displayed by oligopeptide phage clones screened in capture ELISA. Information is given on the peptides identified and the frequencies at which they were found.

No	No. Repeat	Identification	Locus	Peptide Position
1	5	hypothetical protein LEP1GSC042_0155 [*Leptospira kirschneri* serovar Bim str. PUO 1247]	WP_042710668	35–52
2	4	hypothetical protein LEP1GSC112_0402, partial *[Leptospira interrogans* serovar Pomona str. UT364]	EMO00657	17046
3	3	hypothetical protein LEP1GSC124_0783, partial [*Leptospira interrogans* serovar Pyrogenes str. 200701872]	WP_061236626	274–322
4	3	hypothetical protein [*Leptospira interrogans*]	WP_017853878	131–177
5	3	peptidase, M48 domain protein [*Leptospira interrogans* serovar Canicola]	WP_082285734	514–554
6	2	hypothetical protein [*Leptospira kirschneri*]	WP_082292950	1231–1243
7	2	permease [*Leptospira alexanderi*]	WP_078128696	152–167
8	2	glucose-1-phosphate cytidylyltransferase *[Leptospira kirschneri*]	WP_004763753	87–126
9	2	AraC family transcriptional regulator [*Leptospira fainei*]	WP_016550011	239–266
10	1	PBP1A family penicillin-binding protein [*Leptospira interrogans*]	WP_061286131	685–724
11	1	glycosyltransferase [*Leptospira sp*. P2653]	WP_083867789	13–29
12	1	DUF541 domain-containing protein [*Leptospira kirschneri*]	WP_016560806	51–67
13	1	arylsulfatase *[Leptospira fainei*]	WP_016550521	530–550
14	1	sterol desaturase family protein [*Leptospira*]	WP_100784316	242–260
15	1	hypothetical protein LEP1GSC150_4200 [*Leptospira interrogans* serovar Copenhageni str. LT2050]	EMG21699	129–360
16	1	outer membrane protein, TIGR04327 family [*Leptospira fainei*]	WP_016551048	239–259
17	1	flagellar filament outer layer protein Flaa [*Leptospira mayottensis*]	WP_002746704	29–100
18	1	MFS transporter, partial *[Leptospira interrogans*]	WP_025176752	0–80

After three rounds of panning, screening ELISA were performed on phage clones derived from both libraries. Monoclonal phage clones displaying oligopeptides were captured in the wells of a microtiter plate using a monoclonal anti-M13 antibody and screened for reactivity with pooled sera from 18 patients with acute leptospirosis or from 10 healthy donors and detected with a goat anti-human HRP conjugate (Fig 1).

The BLAST results of 18 unique oligopeptide phage clones corresponded to amino acid sequences of peptides consisting of 13–80 amino acids. Six clones corresponded to hypothetical proteins of unknown function; of these, the most frequent one was LEP1GSC042_0155, which was identical in five clones. Altogether, 18/35 clones (51%) corresponded to hypothetical proteins, which agrees with the report that around 40% of genes of *L*. *interrogans*, *borgpetersenii* and *biflexia* encode proteins of unknown function [34]. The peptide fragments of known proteins included various enzymes, transporter proteins, and outer membrane protein.

### Validation of immunogenicity of the synthetic peptides

The selected 18 oligopeptide phage clones produced as monoclonal phage were tested in ELISA using serial dilutions of pooled sera reactive with Malaysian strains, WHO strains and from healthy donors as described above. The peptides of the five most promising phage clones were then synthesized as biotinylated peptides and tested for immunoreactivity against 16 positive and 16 negative individual sera which were not included in the aforementioned two serum pools. The two most abundant proteins in pathogenic *Leptospira spp*., i.e. LipL32 [35] and Loa22 [36], were used as controls.

### Assessment of immunoreactivity and diagnostic accuracy of the selected peptides

**Fig 2:**
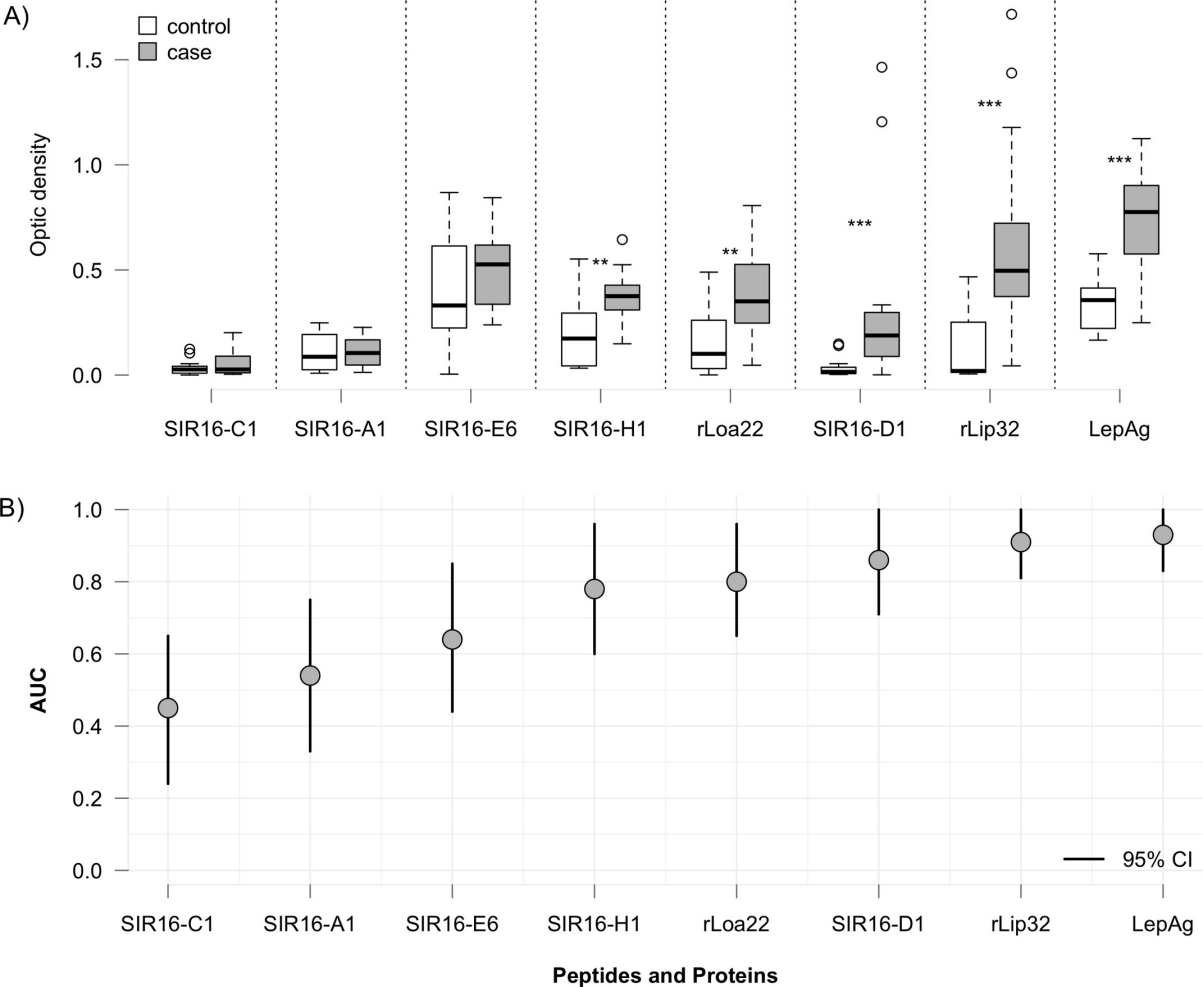
**(A)** Box plot showing seroreactivity of five peptides, two reference proteins (rLoa22, rLipL32) and *Leptospira* culture supernatant antigen against sera from healthy controls of European ethnicity and Malaysian patients with acute leptospirosis (n = 16 each). Serum dilution,1:200. **(B)** Area under the ROC curve (AUC) values of the five peptides and the three references antigens (rLoa22, rLipL32, and *Leptospira* culture antigens). Peptides SIR16-H1 and SIR16-D1 demonstrate discriminatory ability comparable to the reference proteins. The vertical lines delineate 95% confidence intervals (CI). Stars indicate statistically significant difference between case and control groups; **p<0.01, ***p<0.001.

Based on the signal to noise ratio in the screening ELISA and the BLAST analysis, five peptides (SIR16-A1, SIR16-C1, SIR16-D1, SIR16-E6, SIR16-H1; summarized in Table 3) were selected for further validation. Of note, all five selected peptides were derived from the Malaysian genomic library. A validation test was carried out by a titration ELISA (1:200) using individual sera from 16 patients with acute leptospirosis and 16 healthy donors (Fig 2). The aforementioned two recombinant leptospiral reference proteins, rLipL32 and rLoa22, were included for comparison. The results were also compared to *Leptospira* culture supernatant antigen used for in-house ELISA. Two peptides (SIR16-D1, SIR16-H1), the two reference proteins, and the leptospiral antigen reacted significantly more strongly with the patient sera than with the control sera, indicating high immunoreactivity (Fig 2A). In addition, signal strength was comparable among the two peptides and reference proteins.

**Table 3:** Details of seroreactive peptides selected by titration ELISA. Titration ELISA was performed on oligopeptide phage clones using pooled sera reactive with Malaysian strains, WHO strains, and from healthy donors as detailed in S2 Fig.

No. in Table 2	Clone	Blastn	Full length Protein	Peptide Sequence
10	SIR16-A1	*Leptospira interrogans* serovar Copenhageni strain FDAARGOS_203 chromosome	PBP1A family penicillin-binding protein	KSSISLGRGQAASVLAVPIWGRMYNRFYGGQNYPSFGED
7	SIR16-C1	*Leptospira interrogans* serovar Hardjo-prajitno strain Hardjoprajitno chromosome 2	Permease	FGWNRDHFYLDGFFGSY
8	SIR16-D1	*Leptospira interrogans* serovar Manilae strain UP-MMC-NIID HP chromosome 1	Glucose-1-phosphate cytidylyltransferase	NPTAEDWEVDLVDTGALTMTGGRLLRLKDQLSKETFMVTY
14	SIR16-E6	*Leptospira interrogans* serovar Hardjo-prajitno strain Hardjoprajitno chromosome 1	Sterol desaturase	EEPIYGLTKPVTTFDPVYT
4	SIR16-H1	*Leptospira interrogans* serovar Naam str. Naam ctg7180000007595	Hypothetical protein	EFSKTIVEKANQFWMMVRGEGAYSKPTRISQFSIQGLMREEDVLKTS

ROC curve analysis revealed that the two peptides (SIR16-H1 and SIR16-D1) had AUCs close to or greater than 0.8 and thus demonstrated potential as diagnostic biomarkers to differentiate between acute leptospirosis and controls (Fig 2B).

Logistic regression was then used to evaluate whether combinations of the peptides with each other and / or with the two recombinant reference proteins would improve classification. First, using a simulation by resampling from the existing dataset [37] we made a posteriori power analysis for the logistic regression model, which includes four predictors and has the highest AUC. For the 32 samples used in the analysis, we detected a power of 98.9% at a significance level 0.001. Among the 5 peptides, a combination with better classification than the best single peptide could not be identified (Table 4, row 3). This was unexpected, as the reactivity with the individual sera correlated only weakly among the peptides, suggesting that there would be diagnostic synergy (S3 Fig). However, when combining the 5 peptides with the two reference proteins, a classifier consisting of the two best peptides, rLipL32 and the peptide SIR16-A1 was identified that possessed near perfect (AUC, 0.98) discrimination between patient and control sera (Table 4, rows 4 and 6). When combining only the two best peptides with the reference proteins, classification was somewhat less accurate (AUC 0.93 vs. 0.98; compare rows 12 and 14 with 4 and 6), demonstrating the added value of including peptide SIR16-A1. Inspection of the data then revealed that SIR16-A1 had a lower reactivity with two of the control sera, likely explaining the observed improved classification in this multiple regression model.

**Table 4:** Diagnostic performance parameters of single peptides, peptide combinations, and combinations of peptides with reference proteins. Results displayed based on binary comparison of leptospirosis versus healthy donors. Data are based on trade-off values in the ROC curve for each classifier.

No.	Set	Best combination	AUC	P value*	Accuracy^	Sensitivity	Specificity	PPV	NPV
			(95% CI)						
1	Individual peptide	D1	0.85 (0.70–1.0)	3.03e-04	0.844	0.875	0.813	0.824	0.867
2	Individual peptide	H1	0.78 (0.59–0.96)	5.47e-03	0.812	0.938	0.688	0.750	0.917
3	5 peptides	D1	0.85 (0.7–0.98)	3.02e-04	0.844	0.875	0.813	0.824	0.867
4	5 peptides + rLipL32	A1+D1+H1+ rLipL32	0.98 (0.77–1.0)	1.83e-08	0.969	1.0	0.938	0.941	1.000
5	5 peptides + rLoa22	A1+ Loa22	0.96 (0.66–1.0)	1.86e-07	0.938	0.875	1.00	1.00	0.889
6	5 peptides + rLipL32+ rLoa22	A1+D1+H1+ rLipL32	0.98 (0.77–1.0)	1.83e-08	0.969	1.0	0.938	0.941	1.000
7	D1+H1	D1	0.85 (0.70–0.98)	3.02e-04	0.844	0.875	0.813	0.824	0.867
8	D1 + rLipL32	LipL32	0.91 (0.81–1.0)	1.67e-05	0.844	0.938	0.750	0.789	0.923
8	D1 + rLoa22	D1	0.85 (0.70–0.98)	3.02e-04	0.844	0.875	0.813	0.824	0.867
10	H1 + rLipL32	LipL32	0.91 (0.81–1.0)	1.67e-05	0.844	0.938	0.750	0.789	0.923
11	H1+ rLoa22	Loa22	0.80 (0.65–0.96)	2.42e-03	0.781	0.813	0.750	0.765	0.800
12	D1 + H1+rLipL32	D1 + H1+rLipL32	0.93 (0.82–1.0)	3.54e-06	0.938	0.875	1.00	1.00	0.889
13	D1 + H1+ rLoa22	D1 + H1+ rLoa22	0.85 (0,69–1.0)	2.5e-04	0.844	0.687	1.00	1.00	0.762
14	D1 + H1+ rLipL32+rLoa22	D1 + H1+ rLoa22+rLipL32	0.93 (0.82–1)	2.66e-06	0.938	0.875	1.00	1.00	0.889

AUC, area under ROC curve; CI, confidence interval; PPV, positive predictive value; NPV, negative predictive value.

* Uncorrected asymptotic P values.

^ sensitivity + specificity / 2

## Discussion

ORFeome phage display has been proven to be a successful method for selection of immunogenic peptides to be used for diagnostic purposes. It has previously been used successfully to identify novel biomarkers from *Salmonella typhimurium*, *Mycoplasma pneumoniae* and *Neisseria gonorrhea* [14, 20, 38, 39]. Regarding *Leptospira*, it has been used successfully to identify (1) host proteins that interact with LipL32 [40], (2) LigB protein acting as heparin binding protein [41], (3) adhesin activity of *Leptospira* lipoprotein [42], and (4) mimotopes from monoclonal antibodies specific for *Leptospira* spp. [43]. Our study is the first to use phage display to identify immunogenic *Leptospira* antigens from *Leptospira* spp. genomes.

The findings of this study are of particular interest in that they indicate that ORFeome phage display can be used to identify novel peptides for development of leptospiral diagnostics, an approach that promises to be superior to protein or cell extract based methods for use in tropical and resource-limited settings for the reasons outlined in the Introduction. For instance, an ELISA with directly immobilized peptide would constitute a simple peptide-based point-of-care diagnostics for resource-limited settings [44–47]. A very simple antibody-antigen diagnostic based on specially treated paper to immobilize the antigen and a drinking straw as an incubation chamber has been developed for use in resource-limited setting [48] and might constitute an attractive basis for diagnostics based on peptides identified by ORFeome phage display such as ours.

Even though the two identified peptides demonstrated good diagnostic performance, even higher accuracy would be desirable for clinical application. Combinations of these peptides with proteins of immunodominant properties, such as LipL32 may result in better coverage of pathogenic serovars. However, adding recombinant proteins to the assay would obviate the clear advantages of using peptide-based assays. Thus, to preserve the “peptide only” aspect of a new diagnostic, it will be important to assess the diagnostic value of the two dominant antigenic epitopes of LipL32 [49] in peptide form in order to assess whether combining them with our peptides would be useful. In addition, future work should include additional screens to identify other immunoreactive peptides that could synergize diagnostically with SIR16-D1 and/or SIR16-H1 or be superior by themselves.

Since our goal was to identify peptides for the diagnosis of leptospirosis in the acute phase, we evaluated the peptides for reactivity against IgM only. All patient sera were obtained from patients who presented to the health care system after an average of three to five days of illness symptoms. IgM is the first agglutinating antibody to develop 5 to 14 days after exposure to infection and diagnostically meaningful IgG levels appear 1–3 weeks later [9]. It would now be interesting to test whether these peptides are also reactive with IgG and could therefore be used for seroepidemiological surveys, in addition to diagnostics in the acute phase.

It came as a surprise that all five selected peptides were derived from the Malaysian genomic library, but none from the WHO reference genomic library. This is probably because the Malaysian strains had undergone a smaller number of passages in culture than the WHO strains, thus preserving antigen profiles more closely resembling natural infection. This observation has important implications for future work, as it clearly suggests that early passage strains would constitute a better source for ORFeome phage display libraries than extensively subcultured reference strains.

Peptide SIR16-H1 corresponded to a predicted protein of unknown function. In contrast, peptide SIR16-D1 turned out to be a fragment of Glucose-1-phosphate cytidylyltransferase, also known as CDP-glucose pyrophosphorylase, the product of the *rfbF* gene [50–52]. This protein is found in 10 pathogenic leptospiral species and also in some other bacteria. Even though this is an intracellular protein, it is quite feasible that it does lead to a humoral immune response as it might become exposed to the immune system during lysis of leptospirae, during phagocytosis by antigen-presenting cells, or even by being secreted from live leptospirae in the sense of a “moonlighting protein” [53].

This study was limited by the number of sera selected for ELISA and also by the prevalence of serovars in two endemic regions in Malaysia, i.e. Kota Bharu and Kota Kinabalu. Besides, the control group was recruited from healthy donors from a non-endemic region. This is because individuals who had been exposed to leptospirosis in endemic region can produce antibodies from the memory pool, leading to background reactivity and false positive results. In fact, it was reported that healthy individuals in high endemic region have a 15% prevalence of positive anti-*Leptospira* antibodies detected by MAT [54]. Evaluation of the peptides with sera from patients infected with other known tropical infection diseases such as Dengue Fever, Chikugunya, typhoid etc. should be included, as it is important to assess their practicality and specificity as a leptospirosis diagnostic assay in populations exposed to pathogens that may cause serological cross-reactivity. A more comprehensive study involving sera from patients and healthy individuals from various endemic and non-endemic countries should be included in the future, as the present study is based on patient sera from a limited geographical region in Malaysia only and serological responses to field isolates from other geographic areas may differ. All the samples used here were obtained from hospitalized leptospirosis patients, but the immune response may be different in mild presentations. As there are various serovars causing leptospirosis worldwide, we suggest to apply ORFeome phage display screening to genomically more diverse isolates and to human sera collections from various endemic regions for a more universal selection and characterization of the antigen repertoire.

In summary, we report the first study of seroreactive peptides identified by a phage display approach using a combination of endemic *Leptospira spp*. in Malaysia. The synthetic peptides SIR16-D1 and SIR16-H1 showed good potential for the discrimination of acute phase leptospirosis and healthy patients and can form the basis for the development of peptide-based diagnostics for use in resource-limited settings and hot climates.

## Supporting information

S1 Fig Evolution of phage titers during 3 panning rounds with two *Leptospira spp*. genomic libraries. Library 1, Malaysian strains. Library 2, WHO reference strains.
(TIF)

S2 Fig Titration ELISA to assess immunoreactivity of five selected oligopeptide phage clones. Pools from Malaysian patients with acute leptospirosis, which were classified by MAT according to their reactivity with circulating Malaysian strains (Malaysian Pool, n = 8), and WHO reference strains (WHO Pool, n = 10). A pool of non-reactive sera from healthy donors from Germany (Control Pool, n = 8) was used as control.
(TIFF)

S3 Fig Correlations among the five selected peptides and the two reference proteins in terms of immunoreactivity (assessed by ELISA) with the patient and control sera. Data were based on the results shown in Fig 2. Values correspond to Pearson correlation coefficient. Only low correlation is detected between the two best peptides, SIR16-D1 and SIR16-H1.
(TIFF)

## References

[1] Mwachui MA, Crump L, Hartskeerl R, Zinsstag J, Hattendorf J. Environmental and Behavioural Determinants of Leptospirosis Transmission: A Systematic Review. PLoS Negl Trop Dis. 2015;9(9):e0003843 10.1371/journal.pntd.0003843 26379035 PMC4574979

[2] Nascimento ALTO Ko AI, Martins EAL Monteiro-Vitorello CB, Ho PL Haake DA, et al Comparative Genomics of Two Leptospira interrogans Serovars Reveals Novel Insights into Physiology and Pathogenesis. J Bacteriol. 2004;186(7):2164–72. 10.1128/JB.186.7.2164-2172.2004 15028702 PMC374407

[3] Costa F, Hagan JE, Calcagno J, Kane M, Torgerson P, Martinez-Silveira MS, et al Global Morbidity and Mortality of Leptospirosis: A Systematic Review. PLoS Negl Trop Dis. 2015;9(9):e0003898 10.1371/journal.pntd.0003898 26379143 PMC4574773

[4] Lim VK. Leptospirosis: a re-emerging infection. The Malaysian journal of pathology. 2011;33(1):1–5. . 21874744

[5] Garba B, Bahaman AR, Khairani-Bejo S, Zakaria Z, Mutalib AR. Retrospective Study of Leptospirosis in Malaysia. EcoHealth. 2017;14(2):389–98. 10.1007/s10393-017-1234-0 28405850 PMC5486469

[6] Levett PN. Leptospirosis. Clin Microbiol Rev. 2001;14(2):296–326. Epub 2001/04/09. 10.1128/CMR.14.2.296-326.2001 ; PubMed Central PMCID: PMCPmc88975. 11292640 PMC88975

[7] Palaniappan RU, Ramanujam S, Chang YF. Leptospirosis: pathogenesis, immunity, and diagnosis. Curr Opin Infect Dis. 2007;20(3):284–92. Epub 2007/05/02. 10.1097/QCO.0b013e32814a5729 . 17471039

[8] McBride AJ, Athanazio DA, Reis MG, Ko AI. Leptospirosis. Curr Opin Infect Dis. 2005;18(5):376–86. Epub 2005/09/09. . 16148523 10.1097/01.qco.0000178824.05715.2c

[9] Picardeau M, Bertherat E, Jancloes M, Skouloudis AN, Durski K, Hartskeerl RA. Rapid tests for diagnosis of leptospirosis: current tools and emerging technologies. Diagn Microbiol Infect Dis. 2014;78(1):1–8. Epub 2013/11/12. 10.1016/j.diagmicrobio.2013.09.012 . 24207075

[10] Hartskeerl RA SH, Korver H, Goris MGA, Terpstra WJ. International course on laboratory methods for the diagnosis of leptospirosis Amsterdam, the Netherlands: Royal Tropical Institute 2006. Course Manual].

[11] Frenzel A, Schirrmann T, Hust M. Phage display-derived human antibodies in clinical development and therapy. mAbs. 2016;8(7):1177–94. 10.1080/19420862.2016.1212149 27416017 PMC5058633

[12] Crameri R, Kodzius R, Konthur Z, Lehrach H, Blaser K, Walter G. Tapping allergen repertoires by advanced cloning technologies. Int Arch Allergy Immunol. 2001;124(1–3):43–7. Epub 2001/04/18. 10.1159/000053664 . 11306922

[13] GOVARTS C, SOMERS K, HUPPERTS R, STINISSEN P, SOMERS V. Exploring cDNA Phage Display for Autoantibody Profiling in the Serum of Multiple Sclerosis Patients. Ann N Y Acad Sci. 2007;1109(1):372–84. 10.1196/annals.1398.043 17785326

[14] Naseem S, Meens J, Jores J, Heller M, Dübel S, Hust M, et al Phage display-based identification and potential diagnostic application of novel antigens from Mycoplasma mycoides subsp. mycoides small colony type. Vet Microbiol. 2010;142(3):285–92. 10.1016/j.vetmic.2009.09.071. 19900769

[15] Rhyner C, Weichel M, Flückiger S, Hemmann S, Kleber-Janke T, Crameri R. Cloning allergens via phage display. Methods. 2004;32(3):212–8. 10.1016/j.ymeth.2003.08.003. 14962754

[16] Rosander A, Guss B, Frykberg L, Björkman C, Näslund K, Pringle M. Identification of immunogenic proteins in Treponema phagedenis-like strain V1 from digital dermatitis lesions by phage display. Vet Microbiol. 2011;153(3):315–22. 10.1016/j.vetmic.2011.06.005. 21763087

[17] Kügler J, Zantow J, Meyer T, Hust M. Oligopeptide M13 Phage Display in Pathogen Research. Viruses. 2013;5(10):2531 10.3390/v5102531 24136040 PMC3814601

[18] Zantow J, Moreira G, Dubel S, Hust M. ORFeome Phage Display. Methods Mol Biol. 2018;1701:477–95. Epub 2017/11/09. 10.1007/978-1-4939-7447-4_27 . 29116523

[19] Hust M, Meysing M, Schirrmann T, Selke M, Meens J, Gerlach GF, et al Enrichment of open reading frames presented on bacteriophage M13 using hyperphage. Biotechniques. 2006;41(3):335–42. Epub 2006/09/23. 10.2144/000112225 . 16989094

[20] Kügler J, Nieswandt S, Gerlach GF, Meens J, Schirrmann T, Hust M. Identification of immunogenic polypeptides from a Mycoplasma hyopneumoniae genome library by phage display. Appl Microbiol Biotechnol. 2008;80(3):447 10.1007/s00253-008-1576-1 18636254

[21] Becker M, Felsberger A, Frenzel A, Shattuck WM, Dyer M, Kugler J, et al Application of M13 phage display for identifying immunogenic proteins from tick (Ixodes scapularis) saliva. BMC Biotechnol. 2015;15:43 Epub 2015/05/31. 10.1186/s12896-015-0167-3 ; PubMed Central PMCID: PMCPmc4449557. 26024663 PMC4449557

[22] Zantow J, Just S, Lagkouvardos I, Kisling S, Dubel S, Lepage P, et al Mining gut microbiome oligopeptides by functional metaproteome display. Sci Rep. 2016;6:34337 Epub 2016/10/06. 10.1038/srep34337 ; PubMed Central PMCID: PMCPmc5050496. 27703179 PMC5050496

[23] Akmatov MK, Krebs S, Preusse M, Gatzemeier A, Frischmann U, Schughart K, et al E-mail-based symptomatic surveillance combined with self-collection of nasal swabs: a new tool for acute respiratory infection epidemiology. Int J Infect Dis. 2011;15(11):e799–803. Epub 2011/08/20. 10.1016/j.ijid.2011.07.005 . 21852171 PMC7110865

[24] Rondot S, Koch J, Breitling F, Dubel S. A helper phage to improve single-chain antibody presentation in phage display. Nat Biotechnol. 2001;19(1):75–8. Epub 2001/01/03. 10.1038/83567 . 11135557

[25] Soltes G, Hust M, Ng KK, Bansal A, Field J, Stewart DI, et al On the influence of vector design on antibody phage display. J Biotechnol. 2007;127(4):626–37. Epub 2006/09/26. 10.1016/j.jbiotec.2006.08.015 ; PubMed Central PMCID: PMCPmc1866265. 16996161 PMC1866265

[26] Hust M, Dubel S, Schirrmann T. Selection of recombinant antibodies from antibody gene libraries. Methods Mol Biol. 2007;408:243–55. Epub 2008/03/05. 10.1007/978-1-59745-547-3_14 . 18314587

[27] Goris M, Leeflang M, Boer K, Goeijenbier M, van Gorp E, Wagenaar J, et al Establishment of Valid Laboratory Case Definition for Human Leptospirosis. J Bacteriol Parasitol. 2011;3(2). 10.4172/2155-9597.1000132

[28] Bauer DF. Constructing Confidence Sets Using Rank Statistics. Journal of the American Statistical Association. 1972;67(339):687–90. 10.1080/01621459.1972.10481279

[29] Gelman A, Jakulin A, Pittau MG, Su Y-S. A weakly informative default prior distribution for logistic and other regression models. Ann Appl Stat. 2008;2(4):1360–83. 10.1214/08-AOAS191

[30] DeLong ER, DeLong DM, Clarke-Pearson DL. Comparing the Areas under Two or More Correlated Receiver Operating Characteristic Curves: A Nonparametric Approach. Biometrics. 1988;44(3):837–45. 10.2307/2531595 3203132

[31] Zaki MJ, Meira W. Data Mining and Analysis: Fundamental Concepts and Algorithms: Cambridge University Press; 2014.

[32] Altschul SF, Madden TL, Schaffer AA, Zhang J, Zhang Z, Miller W, et al Gapped BLAST and PSI-BLAST: a new generation of protein database search programs. Nucleic Acids Res. 1997;25(17):3389–402. Epub 1997/09/01. ; PubMed Central PMCID: PMCPmc146917. 9254694 10.1093/nar/25.17.3389 PMC146917

[33] Artimo P, Jonnalagedda M, Arnold K, Baratin D, Csardi G, de Castro E, et al ExPASy: SIB bioinformatics resource portal. Nucleic Acids Res. 2012;40(W1):W597–W603. 10.1093/nar/gks400 22661580 PMC3394269

[34] Adler B, de la Peña Moctezuma A. Leptospira and leptospirosis. Vet Microbiol. 2010;140(3):287–96. 10.1016/j.vetmic.2009.03.012. 19345023

[35] Haake DA. Spirochaetal lipoproteins and pathogenesis. Microbiology. 2000;146 (Pt 7):1491–504. Epub 2000/07/06. 10.1099/00221287-146-7-1491 ; PubMed Central PMCID: PMCPmc2664406. 10878114 PMC2664406

[36] Koizumi N, Watanabe H. Molecular cloning and characterization of a novel leptospiral lipoprotein with OmpA domain. FEMS Microbiol Lett. 2003;226(2):215–9. 10.1016/S0378-1097(03)00619-0 14553914

[37] Hosmer D, Lemeshow SJNYJW, Sons. Applied logistic regression. 1989. 1989.

[38] Meyer T, Schirrmann T, Frenzel A, Miethe S, Stratmann-Selke J, Gerlach GF, et al Identification of immunogenic proteins and generation of antibodies against Salmonella Typhimurium using phage display. BMC Biotechnol. 2012;12:29 Epub 2012/06/19. 10.1186/1472-6750-12-29 ; PubMed Central PMCID: PMCPmc3423037. 22703709 PMC3423037

[39] Connor DO, Zantow J, Hust M, Bier FF, von Nickisch-Rosenegk M. Identification of Novel Immunogenic Proteins of Neisseria gonorrhoeae by Phage Display. PLoS One. 2016;11(2):e0148986 10.1371/journal.pone.0148986 26859666 PMC4747489

[40] Chaemchuen S, Rungpragayphan S, Poovorawan Y, Patarakul K. Identification of candidate host proteins that interact with LipL32, the major outer membrane protein of pathogenic Leptospira, by random phage display peptide library. Vet Microbiol. 2011;153(1):178–85. 10.1016/j.vetmic.2011.04.030. 21592685

[41] Ching ATC, Fávaro RD, Lima SS, Chaves AdAM, de Lima MA, Nader HB, et al Lepstospira interrogans shotgun phage display identified LigB as a heparin-binding protein. Biochem Biophys Res Commun. 2012;427(4):774–9. 10.1016/j.bbrc.2012.09.137. 23044419

[42] Lima SS, Ching ATC, Fávaro RD, Da Silva JB, Oliveira MLS, Carvalho E, et al Adhesin activity of Leptospira interrogans lipoprotein identified by in vivo and in vitro shotgun phage display. Biochem Biophys Res Commun. 2013;431(2):342–7. 10.1016/j.bbrc.2012.12.095. 23291183

[43] Tungtrakanpoung R, Pitaksajjakul P, Na-Ngarm N, Chaicumpa W, Ekpo P, Saengjaruk P, et al Mimotope of Leptospira from phage-displayed random peptide library is reactive with both monoclonal antibodies and patients’ sera. Vet Microbiol. 2006;115(1):54–63. 10.1016/j.vetmic.2006.02.011. 16581206

[44] Sagna AB, Gaayeb L, Sarr JB, Senghor S, Poinsignon A, Boutouaba-Combe S, et al Plasmodium falciparum infection during dry season: IgG responses to Anopheles gambiae salivary gSG6-P1 peptide as sensitive biomarker for malaria risk in Northern Senegal. Malar J. 2013;12:301 Epub 2013/08/31. 10.1186/1475-2875-12-301 ; PubMed Central PMCID: PMCPmc3766161. 23988032 PMC3766161

[45] Dubois ME, Hammarlund E, Slifka MK. Optimization of peptide-based ELISA for serological diagnostics: a retrospective study of human monkeypox infection. Vector Borne Zoonotic Dis. 2012;12(5):400–9. Epub 2012/01/06. 10.1089/vbz.2011.0779 ; PubMed Central PMCID: PMCPmc3353756. 22217169 PMC3353756

[46] Dama E, Cornelie S, Camara M, Somda MB, Poinsignon A, Ilboudo H, et al In silico identification of a candidate synthetic peptide (Tsgf118-43) to monitor human exposure to tsetse flies in West Africa. PLoS Negl Trop Dis. 2013;7(9):e2455 Epub 2013/10/03. 10.1371/journal.pntd.0002455 ; PubMed Central PMCID: PMCPmc3784472. 24086785 PMC3784472

[47] Abdel-Fattah M, Charmy R, Tawfik AR. Immune Reactivity of Synthetic Peptides Originated from Hypervariable Region 1 of Hepatitis C Synthetic Consensuses with Egyptian Sera Infected with Hepatitis C Virus Type 4. Intervirology. 2015;58(4):232–41. Epub 2015/09/04. 10.1159/000437385 . 26330263

[48] Chan SK, Lim TS. A straw-housed paper-based colorimetric antibody–antigen sensor. Analytical Methods. 2016;8(6):1431–6. 10.1039/C5AY01828E

[49] Lottersberger J, Guerrero SA, Tonarelli GG, Frank R, Tarabla H, Vanasco NB. Epitope mapping of pathogenic Leptospira LipL32. Lett Appl Microbiol. 2009;49(5):641–5. 10.1111/j.1472-765X.2009.02723.x 19780960

[50] Koropatkin NM, Cleland WW, Holden HM. Kinetic and structural analysis of alpha-D-Glucose-1-phosphate cytidylyltransferase from Salmonella typhi. J Biol Chem. 2005;280(11):10774–80. Epub 2005/01/07. 10.1074/jbc.M414111200 . 15634670

[51] Koropatkin NM, Holden HM. Molecular structure of alpha-D-glucose-1-phosphate cytidylyltransferase from Salmonella typhi. J Biol Chem. 2004;279(42):44023–9. Epub 2004/08/05. 10.1074/jbc.M407755200 . 15292268

[52] Thorson JS, Kelly TM, Liu HW. Cloning, sequencing, and overexpression in Escherichia coli of the alpha-D-glucose-1-phosphate cytidylyltransferase gene isolated from Yersinia pseudotuberculosis. J Bacteriol. 1994;176(7):1840–9. Epub 1994/04/01. ; PubMed Central PMCID: PMCPmc205285. 8144449 10.1128/jb.176.7.1840-1849.1994 PMC205285

[53] Henderson B, Martin AC. Protein moonlighting: a new factor in biology and medicine. Biochem Soc Trans. 2014;42(6):1671–8. Epub 2014/11/18. 10.1042/BST20140273 . 25399588

[54] Reis RB, Ribeiro GS, Felzemburgh RDM, Santana FS, Mohr S, Melendez AXTO, et al Impact of Environment and Social Gradient on Leptospira Infection in Urban Slums. PLoS Negl Trop Dis. 2008;2(4):e228 10.1371/journal.pntd.0000228 18431445 PMC2292260

