# Supplementary figures and images for: Discovery of Leptospira spp. seroreactive peptides using ORFeome phage display
Source: PLoS Negl Trop Dis. 2019 Jan 24;13(1):e0007131. doi: 10.1371/journal.pntd.0007131 (PMC6363232; doi:10.1371/journal.pntd.0007131)

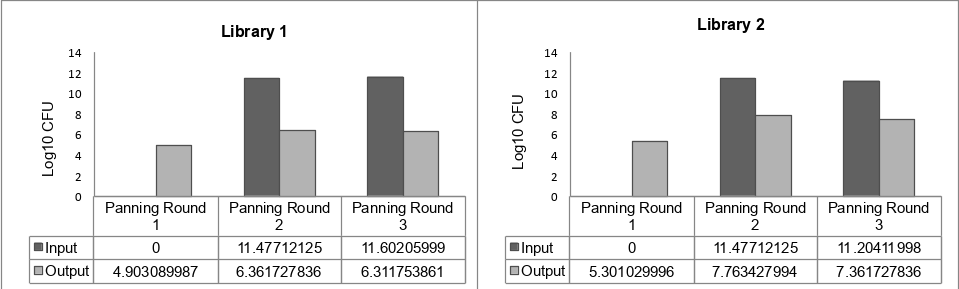

Supplement: S1 Fig — Library 1, Malaysian strains. Library 2, WHO reference strains. (TIF) [file pntd.0007131.s001.tif]

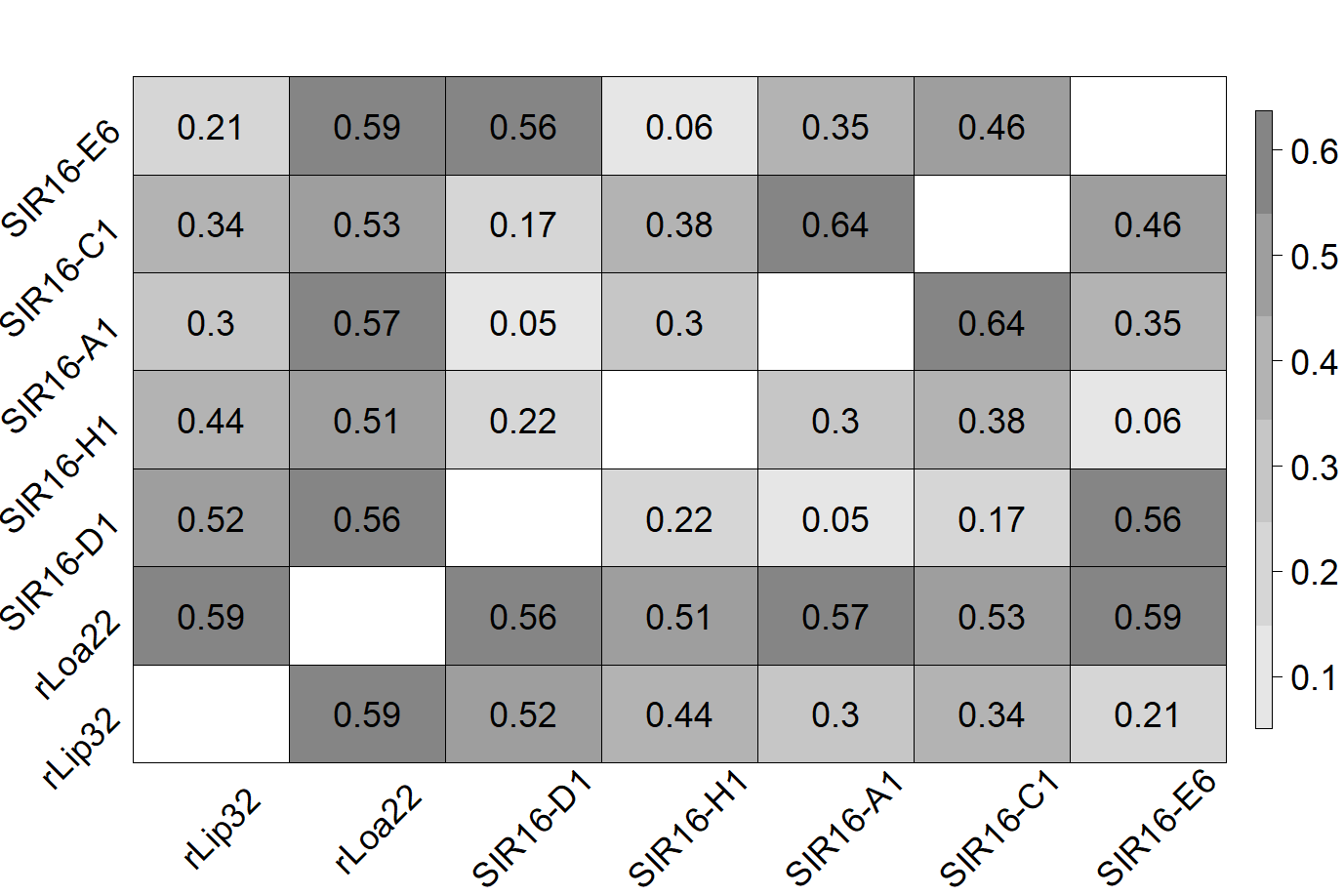

Supplement: S3 Fig — Data were based on the results shown in Fig 2. Values correspond to Pearson correlation coefficient. Only low correlation is detected between the two best peptides, SIR16-D1 and SIR16-H1. (TIFF) [file pntd.0007131.s003.tiff]
